# Synergistic suppression of t(8;21)-positive leukemia cell growth by combining oridonin and MAPK1/ERK2 inhibitors

**DOI:** 10.18632/oncotarget.18503

**Published:** 2017-06-16

**Authors:** Pavel Spirin, Timofey Lebedev, Natalia Orlova, Alexey Morozov, Nadezhda Poymenova, Sergey E. Dmitriev, Anton Buzdin, Carol Stocking, Olga Kovalchuk, Vladimir Prassolov

**Affiliations:** ^1^ Engelhardt Institute of Molecular Biology, Russian Academy of Sciences, Moscow 119991, Russia; ^2^ Belozersky Institute of Physico-Chemical Biology, Lomonosov Moscow State University, Moscow 119992, Russia; ^3^ Dmitry Rogachev Federal Research Center of Pediatric Hematology, Oncology and Immunology, Moscow 117997, Russia; ^4^ National Research Centre “Kurchatov Institute”, Centre for Convergence of Nano-, Bio-, Information and Cognitive Sciences and Technologies, Moscow 123182, Russia; ^5^ Department of Stem Cell Transplantation, University Medical Center Hamburg-Eppendorf, Hamburg 20246, Germany; ^6^ OncoFinder Ltd, Lethbridge, AB T1K7×8, Canada; ^7^ Department of Biological Sciences, University of Lethbridge, Lethbridge, AB T1K3M4, Canada

**Keywords:** acute myeloid leukemia, signaling pathways, RUNX1-ETO, ERK2-inhibitors

## Abstract

One of the most common chromosomal translocations in acute myeloid leukemia is t(8;21)(q22;q22), which results in the appearance of abnormal transcripts encoding for the fusion protein RUNX1-ETO. Therefore, this oncoprotein is considered to be a pertinent and promising target for treating t(8;21) leukemia. Previously, we have shown that downregulation of RUNX1-ETO leads to activation of intracellular signaling pathways enhancing cell survival and determined that the protein ERK2 can mediate activation of most of these pathways. Here we used a combination of oridonin (natural tetracycline diterpenoid), which has been shown to exhibit anti-RUNX1-ETO activity, and ERK2 kinase inhibitors. We found that treatment of leukemic t(8;21)-positive Kasumi-1 cells with oridonin cause decrease of phosphorylated ERK1/2. Treatment of these cells with ERK2 inhibitors makes them more sensitive to RUNX1-ETO inhibition with oridonin. Therefore we postulate that simultaneous inhibition of RUNX1-ETO and ERK2 cause synergistic effect on survival of leukemic cells.

## INTRODUCTION

Acute myeloid leukemia (AML) is a heterogeneous clonal disorder of hematopoietic progenitor cells. The t(8;21)(q22;q22)-derived fusion protein RUNX1-ETO (RE) is present in 12% of de novo AML cases and up to 40% of the AML M2 subtype (FAB classification) [1, 2].

RE affects normal RUNX1-dependent transcrip-tional activation through various dominant-negative mechanisms [3–5]. One of the most important mechanisms is its interaction with nuclear corepressors N-CoR and Sin3A, which recruit the histone deacetylases, leading to a lower level of histone acetylation and less-accessible chromatin [6–8]. It also leads to the suppression of a number of RUNX1 target genes and disruption of mechanisms responsible for normal differentiation of hematopoietic stem cells, such as upregulation of COX/β-catenin signaling pathway, abnormal c-jun pathway activation, and C-KIT pathway hyperactivation [4, 9–11]. Selective suppression of RE leads to a widespread reversal of epigenetic reprogramming and a genome-wide redistribution of RUNX1 binding that results in the inhibition of AML cell proliferation, self-renewal and the induction of differentiation [12].

Targeting of t(8;21)–positive tumor cells has been studied *in vitro* and *in vivo*, and the suppression of AML cells growth by RE inhibiting agents has been recently reported [12–15], however, the effectiveness of such approaches is not obvious. Relapse incidence in t(8;21) patients after standard therapy may reach up to 40% and fusion transcript *RE* is used as a well-established marker for minimal residual disease (MRD) monitoring [16, 17]. Also, in our previous work, the compensatory activation of a wide range of anti-apoptotic and promitotic pathways was shown in experiments with model cell lines, which escape cell death after shRNA silencing of *RE* [13]. We identified that the protein ERK2 (MAPK1), one of the regulators responsible for normal and tumor cell proliferation [18, 19], can mediate activation of 79% of these pathways in viable RE-inhibited cells, as shown by the deep molecular pathway analysis using the OncoFinder method [20, 21]. We proposed the hypothesis that ERK2 associated signaling allows some of the leukemic cells to escape cell death after RE-downregulation.

Oridonin is a diterpenoid isolated from the medicinal herb *Isodon rubescens* and has been shown to be an effective proliferation inhibitor in a wide variety of cancer cells [22–27]. Oridonin was described as an agent that may induce the suppression of proliferation through the induction of p38 MAPK signaling of colon cancer cells [28, 29] and mitochondria- and caspase-dependent apoptosis of the osteosarcoma cell line [30, 31]. Furthermore, a negative effect on tumor growth in nude mice inoculated with t(8;21)-harboring Kasumi-1 cells has also been demonstrated [15, 22]. The anti-leukemia potential of this agent is conferred by its ability to indirectly target the RE oncoprotein, causing its enzymatic cleavage to generate a truncated ΔRE that interacts with RE and interferes with the transregulatory functions of the remaining RE oncoprotein [32]. In this study we aimed to investigate the effect of oridonin in combination with ERK2 inhibitors on leukemic cells to evaluate a possible synergistic anti-survival activity of the two drugs. Here we provide evidence for a synergistic effect in the anti-tumor activity of the oridinin/ ERK-inhibitors combination against t(8;21)-positive AML cells.

## RESULTS

### Choosing effective concentrations of oridonin and ERK2 inhibitors

**Figure 1:**
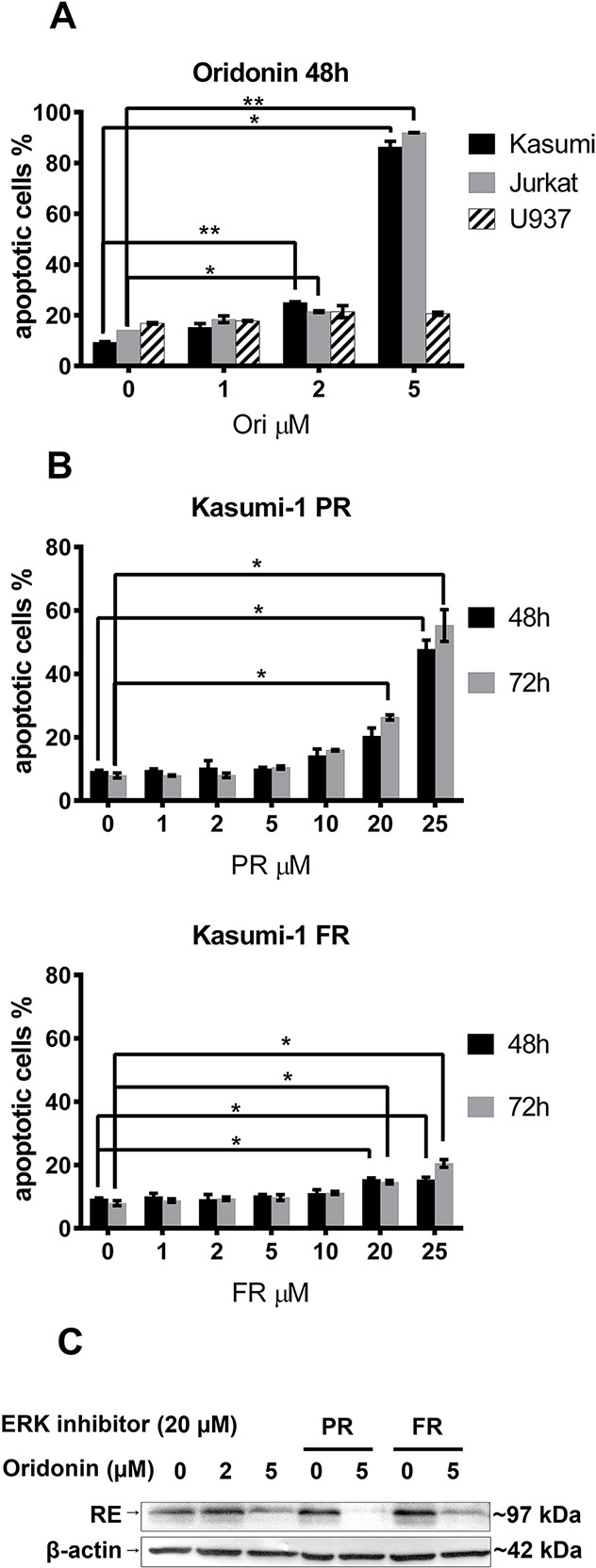
Titration of oridonin and ERK2 inhibitors on Kasumi-1, Jurkat and U937 cells **(A)** Percentage of apoptotic Kasumi-1, Jurkat and U937 cells after 48-hour exposure to various concentrations of oridonin (0-5 μM). **(B)** Percentage of apoptotic Kasumi-1 cells after 48-hour and 72-hour exposure to various concentrations of PR and FR. * P<0.05 and ** P<0.01 represents significant differences compared with according controls. **(C)** Western blotting analysis of the RUNX1-ETO (RE) levels in Kasumi-1 cells in 24 hours after treatment with oridonin (2μM and 5μM concentrations) and ERK inhibitors (20 μM of PR or FR). β-actin was used as loading control.

To detect oridonin-induced cell death, Kasumi-1 cells were treated with a range of concentrations of oridonin (0 to 5 μM) or ERK inhibitors (pyrazolylpyrrole (PR) and pyrazolopyridazinamine (FR) 0 to 25 μM)) diluted in DMSO in parallel with a DMSO containing medium to control for a nonspecific DMSO effect. After 48h and 72h, the percentage of apoptotic cells was measured using flow cytometry. The percentage of Annexin V–positive Kasumi-1, Jurkat, and U937 cells (with exposed phosphatidylserine) depending on the concentration of oridonin or the ERK inhibitor is represented (Figure 1A). At a concentration of oridonin of less than 2 μM, no significant effect on cell viability was detected. The maximum concentration of ERK2 inhibitor PR or FR that did not affect cell survival was 5 μM for PR and 10 μM for FR (Figure 1B).

To detect the oridonin associated inhibition of RE, Kasumi-1 cells were treated with 2 and 5 μM concentrations of oridonin for 24 hours as it was previously published by other group [16]. To exclude the possible influence of ERK inhibitors on oridonin action the maximum concentrations of PR and FR (20 μM) were added. It was shown that oridonin cause suppression of RE levels in Kasumi-1 cells treated with oridonin alone or in presence of ERK inhibitors (Figure 1C).

### Oridonin affects ERK1/2

**Figure 2:**
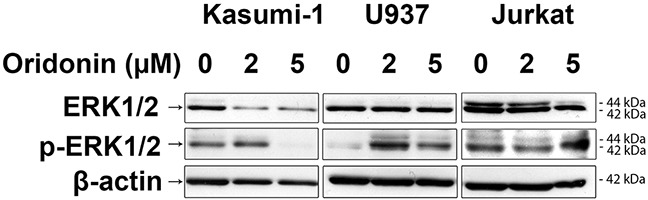
Western blotting analysis of ERK1/2 protein levels Western blotting analysis showing the inactive ERK1/2 levels (two bands 44kDa and 42kDa) in Kasumi-1, U937 and Jurkat cell lines in 24 hours after treatment with oridonin (2μM and 5μM concentrations) and levels of active phosphorylated forms of ERK1/2 (p-ERK1/2). β-actin was used as loading control.

To elucidate the effect of oridonin on levels and ratios of active (phosphorylated) and inactive (non-phosphorylated) ERK1/2 in Kasumi-1, Jurkat and U937 cells we treated them with 2 and 5 μM of oridonin for 24 hours and performed western blot analysis (WB) (Figure 2). We identified that all analyzed cell lines differ by ratios of active and inactive ERK1/2, for example, U937 have very low level of activated ERK1/2 when compared to Kasumi-1 and Jurkat. We found, that treatment of RE-negative cells with oridonin leads to higher levels of phosphorylated ERK1/2 in U937 cells but not in Jurkat cells, in which the level of phosphorylated ERK1/2 does not change comparatively to control. We found that addition of oridonin to RE-negative cell lines (both U937 and Jurkat) cause no changes in the levels of inactive ERK1/2. The opposite effect was found in RE-positive cells. We showed that treatment of Kasumi-1 cells with oridonin cause decrease of both phosphorylated and non-phosphorylated ERK1/2.

### Apoptosis induced by oridonin, ERK2-inhibitors and their combination on RE-positive cells

**Figure 3:**
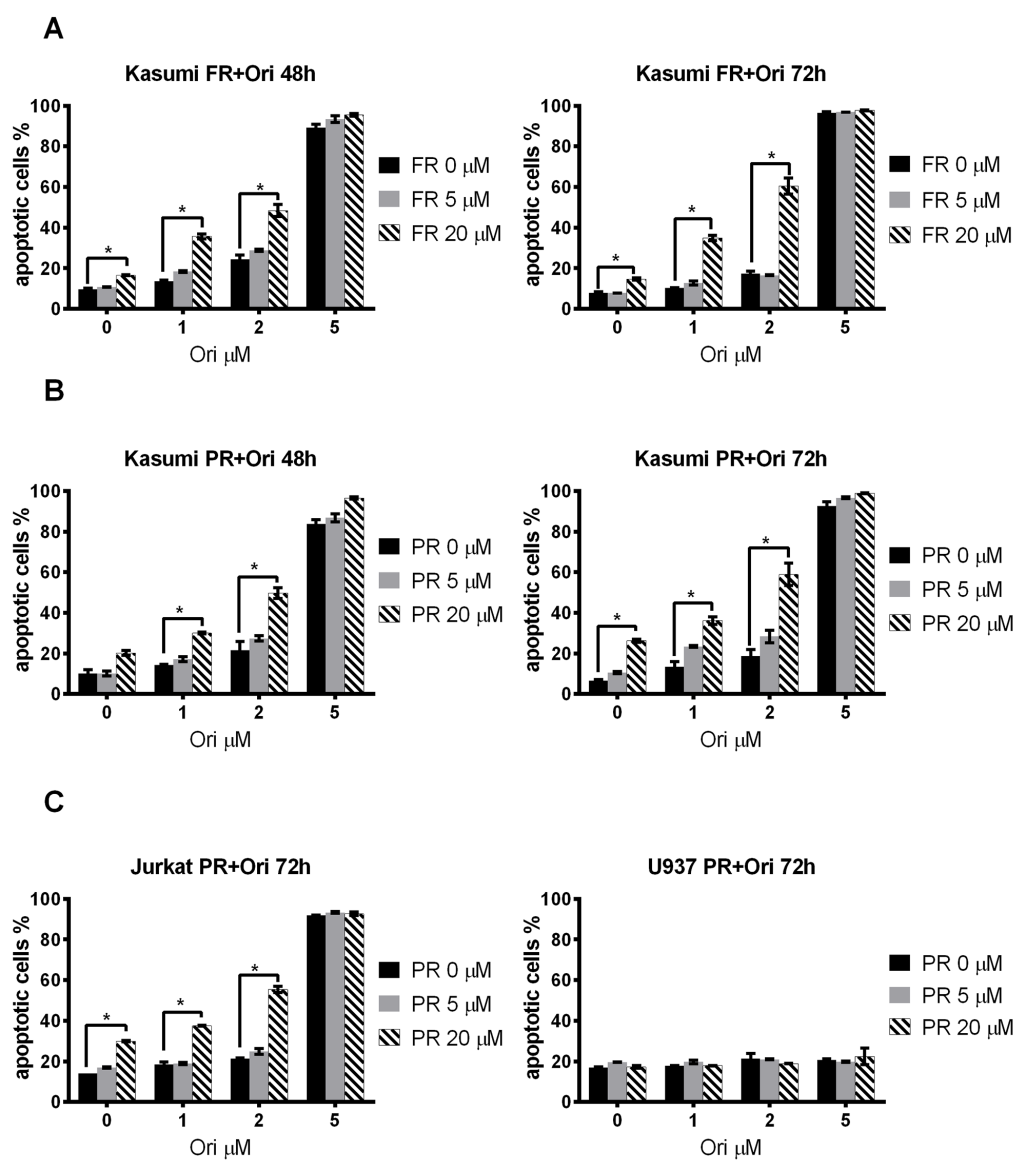
Effect of oridonin and ERK2 inhibitors on apoptosis in Kasumi-1, Jurkat and U937 cells **(A)** Dose-dependent effect of FR on Kasumi-1 cells apoptosis after 48-hour and 72-hour exposure to the inhibitor in presence of different concentrations of oridonin (0-5 μM). * P<0.05 represent significant differences as compared to controls without oridonin for each concentration of FR. **(B)** Dose-dependent effect of PR on Kasumi-1 cells apoptosis after 48-hour and 72-hour exposure to inhibitor in presence of different (0-5 μM) concentrations of oridonin. * P<0.05 represents significant differences compared with controls without oridonin for each concentration of PR. **(C)** Dose-dependent effect of PR on Jurkat and U937 cells apoptosis after 72-hour exposure to inhibitor in presence of different concentrations of oridonin. * P<0.05 represents significant difference as compared to controls without PR for each concentration of oridonin.

Next we measured an impact of oridonin on the apoptosis of t(8;21)-positive AML Kasumi-1 cells when added in combination with ERK inhibitors. The percentage of Annexin V–positive Kasumi-1 cells (with exposed phosphatidylserine) in the presence of the combination of oridonin and the ERK2 inhibitor PR or FR was significantly higher than in cells with these inhibitors added alone. The most pronounced effect was detected at 72h in a combination of 2 μM oridonin and 20 μM FR from 15% or 17% in control cells (0 μM oridonin and 20 μM FR or 2 μM oridonin and 0 μM FR, respectively) to ∼53% (Figure 3A). The effect of oridonin in combination with the ERK inhibitor PR was also measured, and a similar effect to that of the addition of FR was noted—the percentage of apoptotic Annexin V–positive cells increased from ∼26% or ∼19% in control cells (0 μM oridonin and 20 μM PR or 2 μM oridonin and 0 μM PR, respectively) to ∼59% (2 μM oridonin and 20 μM PR) (Figure 3B).

The synergistic effect on apoptosis caused by the combination of inhibitors was then calculated (see the Materials and Methods section). We found that oridonin and the ERK2 inhibitors caused synergistic effect on apoptosis of the Kasumi-1 cells. The most prominent effect was found when 2 μM of oridonin and 20 μM of FR were added to t(8;21)-positive cells (Table 1). These data suggest that ERK and oridonin inhibitors added in combination cause a synergistic and not simply an additive effect on the apoptosis of Kasumi-1 cells.

**Table 1:** Synergistic effect of oridonin in combination with ERK2 inhibitors

	Kasumi-1				
Time	ORI	PR		FR	
		5 μM	20 μM	5 μM	20 μM
**48h**	1 μM	2,98±3,62	5,81±3,47	3,63±1,06	15,21±1,56
	2 μM	5,89±5,7	18,2±6,15	3,89±2,34	17,25±3,82
	5 μM	3,07±4,43	2,68±4,03	3,64±2,55	−0,46±1,99
**72h**	1 μM	5,94±2,69	2,89±3,32	2,59±1,28	16,49±2,97
	2 μM	5,57±4,58	20,38±6,54	−0,76±1,53	36,41±4,26
	5 μM	−0,02±2,49	−13,5±2,52	0,47±1,06	−5,55±1,2
		**Jurkat**		**U937**	
**Time**	**ORI**	**PR**		**PR**	
		**5 μM**	**20 μM**	**5 μM**	**20 μM**
**72h**	1 μM	−2,35±1,47	3,3±1,37	−0,85±0,97	−0,3±0,85
	2 μM	0,7±1,43	18,2±1,52	−3,25±2,47	−2,85±2,54
	5 μM	−1,4±0,53	−15,1±0,97	−3,65±0,87	1,4±4,27

Values in the table represent synergistic effect of combinations of oridonin and ERK2 inhibitors (PR and FR) at various concentrations measured after 48h, 72h exposure of Kasumi-1, Jurkat and U937 cell lines to both drugs. These values were calculated according to the formula provided in Materials and Methods section. The higher positive values indicate more pronounced synergistic effect. The negative or close to zero values imply absence of synergy. All values in the table are presented as means and their standard deviations.

### Apoptosis induced by oridonin, ERK2 inhibitors, and their combination on RE-negative cells

The same scheme was used to study the effect of ERK inhibitors/oridonin combination on the apoptosis of t(8;21)-negative Jurkat or U937 cells after 48, 72, and 96 hours of exposure. Oridonin appeared to be highly toxic for Jurkat cells at 5 μM concentration. The maximal effect was detected at the combination of 20 μM PR and 2 μM oridonin—the percentage of Annexin V–positive cells increased from ∼30% (20 μM PR added alone) or ∼21% (2 μM oridonin added alone) to ∼55% when added in combination to the control cells (Figure 3C, Table 1).

No significant change to the number of apoptotic cells compared with the control was detected when the same concentrations of ERK inhibitors and oridonin were added to U937 cells (FAB M4/M5 AML) (Figure 3C). Also, no additive effect on cell death was found when the inhibitors were used in combination.

### Oridonin and ERK2 inhibitors have a synergistic suppressive effect on RE-positive cell survival but not on RE-negative cells

**Figure 4:**
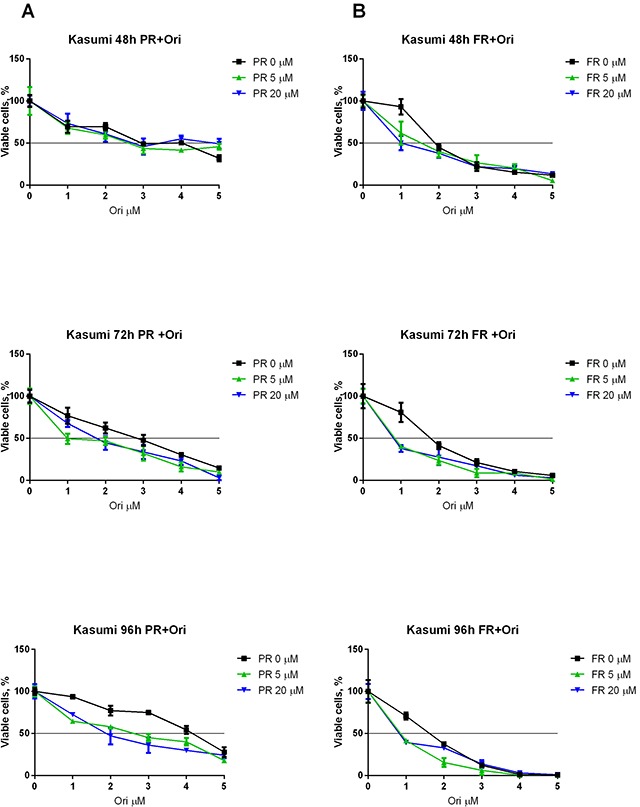
Synergistic effect of oridonin and ERK2 inhibitors on proliferation of Kasumi-1 cells **(A)** Growth curves represent dose dependent effect of oridonin (0-5 μM) on Kasumi-1 cells proliferation after 48, 72, and 96-hour exposure in presence of different concentrations of PR (5 and 20 μM). **(B)** Growth curves represent dose dependent effect of oridonin (0-5 μM) on Kasumi-1 cells proliferation after 48, 72, and 96-hour exposure in presence of different concentrations of FR (5 and 20 μM). In all experiments amount of viable cells treated with ERK-2 inhibitor alone (0; 5 or 20 μM) was taken as 100% for each curve. Black line shows the level where percentage of alive cells reaches 50%.

To elucidate whether a combination of ERK inhibitors and oridonin has a synergistic antiproliferative/proapoptotic effect on the t(8;21)-positive cells, we measured the viability of the Kasumi-1 cells with Trypan blue and the IC_50_ of oridonin combined with different concentrations of the ERK inhibitor PR (Figure 4A) and ERK inhibitor FR (Figure 4B) was calculated. It appeared that supplementing the media with 5 or 20 μM (PR or FR) decreased the IC_50_ of oridonin more than two-fold (Table 2).

**Table 2:** IC50 of oridonin in combination with ERK2 inhibitors

Time	Kasumi-1			Kasumi-1		
	PR 0 μM	PR 5 μM	PR 20 μM	FR 0 μM	FR 5 μM	FR 20 μM
48h	3,22	2,67	3,68	1,93	1,45	1,06
72h	2,59	1,24	1,73	1,75	0,81	0,74
96h	2,38	1,27	2,04	1,51	0,86	0,84
		**Jurkat**			**U937**	
	**PR 0 μM**	**PR 5 μM**	**PR 20 μM**	**PR 0 μM**	**PR 5 μM**	**PR 20 μM**
48h	1,72	1,98	1,26	2,98	>5	>5
72h	1,20	2,24	1,57	2,95	4,66	>5
96h	1,18	1,74	1,09	2,38	>5	>5

Table represent IC50 in μM of oridonin at various concentrations of ERK2 inhibitors (PR and FR) measured after 48h, 72h, 96h exposure of Kasumi-1, Jurkat and U937 cell lines to both drugs.

**Figure 5:**
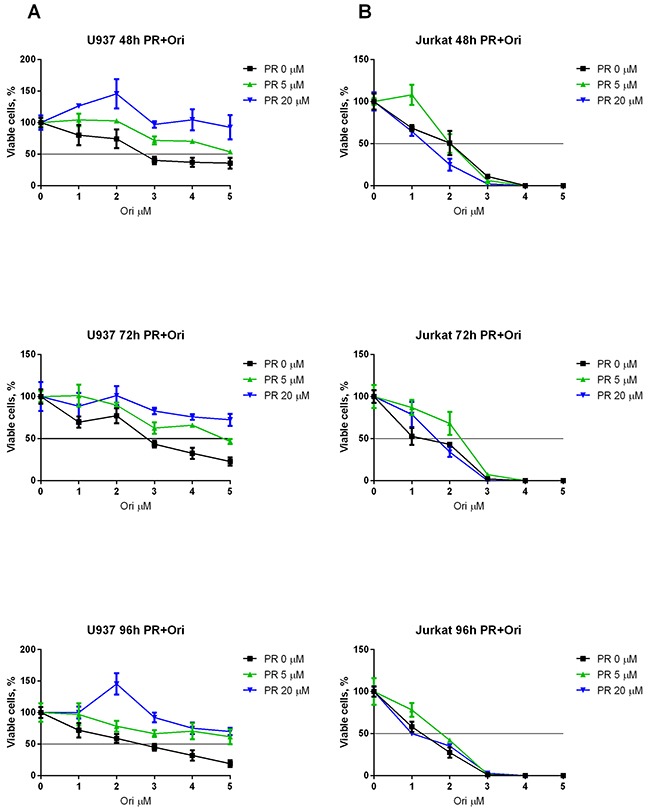
Combined effect of oridonin and ERK2 inhibitors on proliferation of Jurkat and U937 cells **(A)** Growth curves represent dose-dependent effect of oridonin (0-5 μM) on U937 cells proliferation after 48, 72, and 96-hour exposure in presence of different concentrations of PR (5 and 20 μM). **(B)** Growth curves represent dose-dependent effect of oridonin (0-5 μM) on Jurkat cells proliferation after 48, 72, and 96-hour exposure in presence of different concentrations of PR (5 and 20 μM). In all experiments amount of viable cells treated with ERK-2 inhibitor alone (0; 5 or 20 μM) was taken as 100% for each curve. Black line shows the level where percentage of alive cells reaches 50%.

The viability and level of apoptosis in RE-negative Jurkat and U937 cells were also measured. Oridonin displayed less cytotoxicity for the U937 cells than for the Kasumi-1 cells (Figure 5A). Furthermore, we observed a slight increase in viability when the cells were subjected to PR which resulted even in an increased IC_50_ of oridonin. Surprisingly, oridonin displayed higher cytotoxicity for the Jurkat cells than for the Kasumi-1 cells (Figure 5B). However, the ERK inhibitors had no effect on the IC_50_ of oridonin in this cell line (Table 2). Similar results were obtained in experiments with the other ERK inhibitor (FR). As with PR, the maximum effect was observed 72h after the addition of the inhibitors. The use of 5 μM or 20 μM of FR decreased the IC_50_ of oridonin twice in both cases. A decrease in the IC_50_ of oridonin was also observed 48 h after the addition of the inhibitors, but it was not as strong as it was at 72 h (Table 2).

Taking together we found that the combination of oridonin with ERK inhibitors resulted in an increased percentage of apoptotic cells. The effect of the drug combination was stronger than effect of the individual drugs for both PR and FR; therefore, we conclude that this effect is synergistic. It indicates that ERK inhibitors specifically induce apoptosis in RE-inhibition resistant cells when added in combination with oridonin—a drug that has been shown to have specific anti-RE activity.

## DISCUSSION

The aim of this *in vitro* study was to investigate whether the pro-survival effects observed in a population of RE-suppressed AML cells is caused in part by the activation of ERK2-associated signaling pathways. A secondary aim was to determine if potential synergisms with anti-RE and anti-ERK2 agents may be effective in t(8;21)-positive leukemia.

In leukemic cells the oncoprotein RE directly and indirectly disrupt the functions of genes responsible for normal hematopoiesis [6, 7, 12, 33–35]. Cells in which RE was suppressed had decreased growth potential, but on the other side, this study has revealed the potential to escape cell death, probably by activating signaling pathways enhancing cell survival and proliferation. In previous study, it was demonstrated that RE silencing in Kasumi-1 cells indirectly induces the expression of proteins promoting cell cycle progression, such as BCL2; GATA1; TCF12; Sp1; and, most importantly, ERK2 (MAPK1) [13]. ERK2—one of the key regulators responsible for normal and tumor cell proliferation [18, 19]—may alone be responsible for the activation of at least 23 of the promitotic signaling pathways.

**Figure 6:**
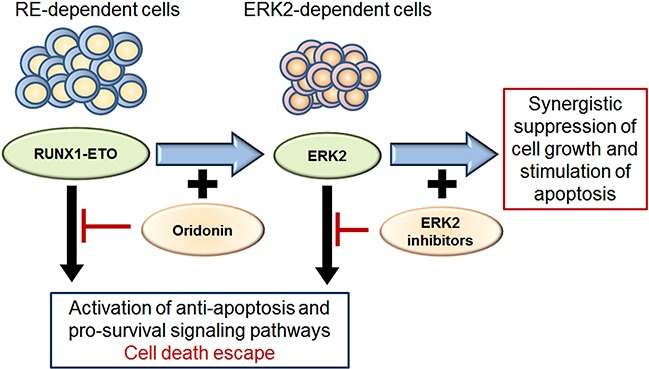
Scheme for ERK2 dependent survival of cells with inhibited RE and effect of ERK2 inhibitors and oridonin Both RE and ERK2 activate pro-survival and anti-apoptotic signaling pathways in leukemic cells. The oridonin-induced inhibition of RE makes leukemic cells dependent on pro-survival mechanisms associated with ERK2/MAPK signaling. Inhibition of ERK2 activity block potential cell death escape mechanisms and renders cells more sensitive to RE inhibition by oridonin. Thus simultaneous inhibition of RE and ERK2 has synergistic suppressive effect on cell growth and apoptosis.

We hypothesized that inhibiting ERK2 could enhance apoptosis in cells with downregulated RE. To test this hypothesis, Kasumi-1 cells were subjected to both an RE inhibitor (oridonin) and to ERK inhibitors (PR and FR) (Figure 6). Both ERK inhibitors significantly decreased viability of oridonin-treated cells, which was confirmed by two different methods (Trypan blue staining and measuring the percentage of apoptotic cells).

We found that when used separately, oridonin or ERK2 inhibitors caused induction of apoptosis in RE-positive Kasumi-1 cells. It is known that RE may cause anti-apoptotic effect through the BCL-2 activation [36]. Also, activation of BCL-2 is tightly associated with ERK1/2 [37]. We identified that Kasumi-1 cells have high level of phosphorylated ERK1/2. Treatment of Kasumi-1 cells with oridonin causes decrease of RE and ERK1/2 levels (both active and inactive forms). Possibly the decrease of ERK1/2 levels in Kasumi-1 cells treated with oridonin may explain the lower IC50 of oridonin when added in combination with ERK inhibitors, because of lower dose of oridonin is necessary for ERK2 inhibition which may be associated with RE downregulation.

Interestingly, exposure of U937 AML cells (FAB M4/M5) to a combination of ERK inhibitor and oridonin had an opposite effect significant increase in the IC_50_ in comparison with control cells in which inhibitors were added alone, and no changes in apoptosis. It is known that oridonin (in absence of RE) may cause activation of ERK1/2 [38].

Taking into account our WBs experiments (Figure 2), and data published by other groups, it is possible that lower sensitivity of U937 to pro-apoptotic effect of oridonin may be associated with ERK1/2 activation and contribution of pro-survival mechanisms, associated with activation of anti-apoptotic protein BCL-2. Though, we have not seen significant increase in apoptosis for U937 cells after oridonin treatment, we observed that oridonin inhibited U937 cell proliferation. Addition of ERK inhibitors suppressed anti-proliferative effect of oridonin on these cells. That may suggest that activation of ERK1/2 suppress U937 proliferation. This may be explained by the fact that BCL-2 has both anti-apoptotic and anti-proliferative effect [39]. Overall our data suggests that in U937 cell line ERK1/2 activation may protect cells from oridonin-induced apoptosis, but has negative effect on cell growth rate.

We observed, that combination of oridonin and ERK2 inhibitors had a synergistic effect on apoptosis when added to Kasumi-1 cells or Jurkat cells. On the contrary, unlike the Kasumi-1 cells, which were more sensitive to the combination of the inhibitors, Trypan blue staining and the analysis of growth showed no changes in IC_50_ of oridonin when added in combination with ERK2 inhibitors to Jurkat or U937 cells. The lack of oridonin associated activation of ERK1/2 in Jurkat cells suggests other mechanisms for apoptosis induction. For example, it was described, that oridonin can target heat shock protein 70 1A (HSP70 1A) and also and also cause blockade of the NF-kappa B signal pathways in Jurkat cells [40, 41].

Here we demonstrated that oridonin, a potential RE-targeting agent, may have a synergistic effect on t(8;21)-positive Kasumi-1 AML cell growth when added to an ERK2 inhibitor. We showed that oridonin treatment has dualistic effect on ERK1/2 and it differs between RE-positive and RE-negative cells. This fact along with our previous results may suggest that specific relation between RE and ERK2 action is present. The combination of ERK inhibitors and oridonin more efficiently can inhibit growth of t(8;21)-positive leukemic cells and may be less toxic for t(8;21)-negative cells, even than oridonin alone.

These results suggest that ERK2 may be one of the key factors responsible for survival of a subpopulation of AML t(8;21)-positive cells with suppressed RE, providing possible therapeutic derivations for resistant tumor cells. Also this study provides a clue for how to make RE-targeted therapy more effective and reduce possible side effects.

## MATERIALS AND METHODS

### Drugs

Oridonin was purchased from Sigma (Sigma-Aldrich Co., St. Louis, USA). pyrazolylpyrrole (PR) and pyrazolopyridazinamine (FR) was purchased from Santa Cruz (Santa Cruz Biotechnology, Dallas, USA).

### Cell culture

Human acute leukemia cells Kasumi-1, carrying the *RE* oncogene and Jurkat were cultured in RPMI 1640 medium supplemented with 20% fetal calf serum (FCS), 100 units/ml penicillin, 100μg/ml streptomycin and 1 mM sodium pyruvate at 37°C and 5% CO_2_. U937 cells were cultured in RPMI medium plus 10% fetal calf serum (FCS), containing 100 units/ml penicillin, 100μg/ml streptomycin and 1 mM sodium pyruvate at 37°C and 5% CO_2_. Kasumi-1, U937, Jurkat cells were obtained from the Heinrich-Pette Institute - Leibniz Institute for Experimental Virology.

### Analysis of apoptosis

The cells were plated into 24 well plate in concentrations of 7*10^4^ cells per well in 1 ml final volume. Apoptosis was measured by double staining with Annexin V-FITC (Molecular Probes) and PI as it was described previously [14]. All measurements were performed on a Beckman Coulter Epix XL4 flow cytometer (Beckman Coulter) and analyzed with WinMDI software. All reported values are means of three independent measurements with standard deviations.

### Analysis of cell growth

The cells were counted by Trypan blue exclusion method. Cells were plated into 96 well plate in concentrations of 7*10^3^ cells per well in 100 μl final volume. 0.04% Trypan blue solution (Gibco by Life Technologies Corp., Grand Island, NY, USA) made in PBS was added to the cells in a 1:1 ratio. Trypan blue dye enters cells with compromised cell membranes while live cells exclude the dye. Non-viable cells appear blue under a bright-field microscope. For each sample, cells were counted to score for % dead cells by Trypan blue uptake.

### Statistical analysis

All the data were expressed as mean ± SEM from three individual experiments. Statistical significance of differences observed in oridonin and ERK inhibitors -treated versus control cells was determined by using an unpaired Student t-test. A p-value of lower 0.05 marks significance.

### Synergism analysis for evaluation of the effect of combining drugs

Oridonin IC50 was calculated from dose-dependent growth curves of Kasumi-1, Jurkat and U937 cells in presence of ERK2 inhibitors. IC50 of oridonin was defined as the concentration of oridonin that results in 50% decrease in number of viable cells. For each concentration of ERK2 inhibitors 100% was defined as number of viable cell without oridonin. Calculation of IC50 was performed using Graph Pad Prism (GraphPad Software, San Diego, CA) with nonlinear regression curve fitting. To calculate the possible synergistic effect caused by used inhibitors on apoptosis of cells we proposed to calculate it as follows:

Total effect = Effect of the combination – Effect of oridonin – Effect of PR/FR.

Effect of combination = Percentage of apoptotic cells observed (combination of oridonin and ERK2 inhibitor) – percentage of apoptotic cells in control sample (when no agents added),

Effect of oridonin = Percentage of apoptotic cells observed (oridonin) – percentage of apoptotic cells in control sample (when no agents added),

Effect of PR/FR = Percentage of apoptotic cells observed (PR/FR) – percentage of apoptotic cells in control sample (when no agents added).

### SDS-PAGE and western blot

Cellular lysates were analyzed in 8 or 10% polyacrylamide gels using Tris-Glycine buffer system and transferred onto nitrocellulose membranes. Primary antibodies were used to detect RE, ERK1/2, p-ERK1/2. Rabbit monoclonal anti-p44/42 MAPK (Erk1/2) antibodies (4695S, Cell Signaling, USA), rabbit monoclonal anti-Phospho-p44/42 MAPK (Erk1/2) (Thr202/Tyr204) (4377S, Cell Signaling, USA, rabbit polyclonal anti-AML1 (4334S, Cell Signaling, USA) antibodies were used in dilution 1:1000. Blots were then incubated with goat anti-rabbit IgG conjugated to horse radish peroxidase (HRP) (Abcam, UK and Enzo, USA) and developed using ECL Prime kit (GE Healthcare, UK). To confirm the equal protein load, the membranes were stripped and stained with anti-β-actin antibodies (Abcam, UK) diluted 1:1000, followed by incubation with goat anti-mouse HRP conjugates (Enzo, USA). The blots were developed as indicated above.
